# Palaeolake isolation and biogeographical process of freshwater fishes in the Yellow River

**DOI:** 10.1371/journal.pone.0175665

**Published:** 2017-04-13

**Authors:** Bin Kang, Xiaoxia Huang, Yunfei Wu

**Affiliations:** 1 Fisheries College, Jimei University, Xiamen, China; 2 School of Earth Science, Yunnan Institute of Geography, Yunnan University, Kunming, China; 3 Fisheries College, Ocean University of China, Qingdao, China; Southwest University, CHINA

## Abstract

The Yellow River, one of the very few in the Earth, originated from many dispersive palaeolakes. Taking this unique advantage, we examined the roles of palaeolake isolation vs. geological processes vs. climate in determining current fish biogeographic pattern. We reviewed available data on fish species and their geographical distribution in the river, as well as palaeolake development, geological and climatic parameters. The 138 fish species recorded in the river could be divided into 8 biogeographic regions, corresponding to the distribution of palaeolakes and respective endemic species. Through variation partitioning analysis, palaeolake isolation was the most influential factor explaining 43.6% of the total variance on the current fish distribution. The Quaternary Ice Age produced a transitional distribution for fishes from the glacier to warm water, especially for the subfamily Schizothoracinae, which showed various degrees of specialisation along altitudes. We suggested that fish biogeography in the Yellow river was basically shaped by palaeolake isolation, and further carved under serials of geologic events and contemporary climate change.

## Introduction

Freshwater fishes are among the most imperilled faunas worldwide under anthropogenic threats [1]. Unfortunately, knowledge about diversity and geography on freshwater fishes remains poor because of ‘the Linnean shortfall’ (most species living on Earth are still not described) and ‘the Wallacean shortfall’ (geographic distributions of most species are poorly understood) [2]. Therefore, analyses using existed data to elucidate the biogeographical pattern and historical process are greatly encouraged.

Palaeogeological events are often considered highly relevant to complex geographical patterns of freshwater fishes than other factors [3, 4], such as ancient river isolation [5], glacierization [6], volcanism [7] and long-term basin boundaries [8], according to ‘history hypothesis’ [9]. On the other hand, contemporary environmental conditions, which support highly diverse microhabitats and opportunities for species adaptation and radiant evolution [10], also act as an important role in determining spatial pattern of species nowadays according to a common assumption that organisms sharing the same geographical location also share the same current environmental constraints [11], defining as ‘environmental niche hypothesis’ [12]. How much of the visible geographical distribution is due to historic process or biotic responses to the current environmental conditions is still the subject of considerable debate [13].

Large rivers, covering a wide range of geological terrain such as latitude, longitude, or altitude, and multiple climate zones, are viewed as separate systems with their respectively historic processes and ecological characteristics [14]. From headwater to estuary, natural lakes, waterfall, swamps and seasonal floodplains serve as possible ‘discontinuities’, dividing rivers into more or less independent parts [15], thus giving a chance to elucidate the isolation of fish distribution patterns. The Yellow River (Huang He), the third longest river in Asia and the seventh longest river in the world, originates from the northeastern margin of the Tibetan Plateau and then flows eastward through the Ordos Plateau and the North China Plain into the Bohai Sea. It is notable for carrying a large amount of silt as 1.6 billion tons annually at the point where it descends from the Ordos Plateau [16]. The Yellow River showed a unique geological process from many scattered palaeolakes. Following a series of next tectonic events, each palaeolake developed the respective water system with distinct gorges, and connected into a large river across the northern China in the end [17]. Fishes migrated or restricted in the channel accompanying with the disappearance or connection of palaeolakes, geologic events as well as the influence of contemporary climate, and gradually formed the current pattern. Fauna there belongs to Palearctic at a global scale, and the source region was even considered as an independently Tibetan Plateau fauna, characterized by the endemic subfamily Schizothoracinae (Cyprinidae) adapting to plateau frigid climate [18]. Unfortunately information on geographic distribution and ecological traits of individual fish species, as well as corresponding environmental conditions in this region are scattered, and no analyse on fish spatial pattern and process in detail is conducted. In this paper, we 1) mapped the current spatial pattern of freshwater fishes along the Yellow River at a taxonomic scale; 2) measured the roles of palaeolake, geology and climate in shaping the fish spatial pattern; and 3) elucidated the processes determining fish diversity and distribution of the Yellow River.

## Materials and methods

### Study area

**Fig 1:**
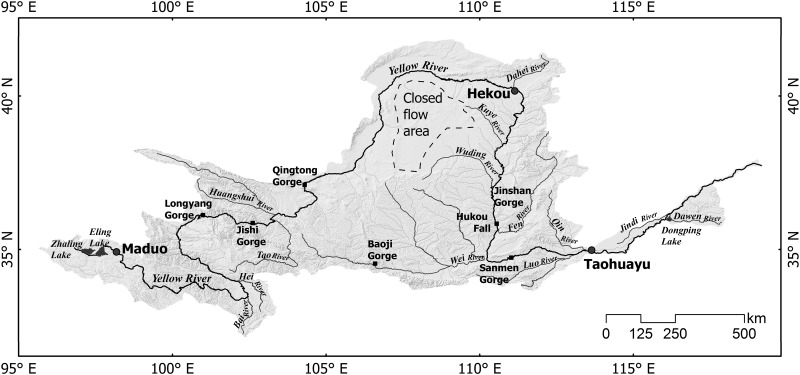
Topographic map of the Yellow River basin. The entire basin is divided into four parts, including the source (from head to Maduo), upper Yellow (from Maduo to Hekou), middle Yellow (from Hekou to Taohuayu) and lower Yellow (from Taohuayu to the river mouth). There is a closed flow area in the Loess Plateau with no water exchange with the Yellow River.

The Yellow River originates in the Bayankala Mountains and winds through 9 provinces of China, namely Qinghai, Gansu, Ningxia, Inner Mongolia, Shaanxi, Shanxi, Henan, and Shandong from west to east in sequence. The river length is 5,464 km; the drainage area is 752, 443km^2^, spanning about 10° of latitude and 23° of longitude (N 32°-42°, E 96°-119°); the average elevation is 1,547 m with the maximum up to 4,800 m. Most of the drainage basin is semi-desert or steppe grasslands with an average annual rainfall to only 300 mm [19]. The source region of the Yellow River ended at Maduo of Qinghai Province. The upper Yellow River constitutes a segment from Maduo to Hekou Town of Inner Mongolia, just before it turns sharply to the south. Along this distance, the elevation drops 3,496 m with an average grade of 0.10%. The middle part is between Hekou and Taohuayu of Henan Province, with a total elevation drop of 890 m and an average grade of 0.074%, and contributes 92% of the river's silts when passing through the Ordos Plateau. Totally 30 large tributaries attach to this part, and the water flow is increased by 43.5% on this stage. The lower reach, from Taohuayu to estuary, is confined to a levee-lined course as flowing eastward across the North China Plain. The total drop in elevation is 94 m, with an average grade of 0.012% (Yellow River Conservancy Commission, http://www.yellowriver.gov.cn/). Important tributaries directly connecting with the mainstream from upstream to downstream are the Bai River, the Hei River, the Tao River, the Huangshui River, the Dahei River, the Kuye River, the Wuding River, the Fen River, the Wei River, the Luo River, the Qin River, the Jindi River and the Dawen River, and the Wei River is the largest tributary (Fig 1).

### Data collection

#### Fish

Taxonomic records of fishes in the Yellow River were mostly derived from historical fishery survey reports, as well as detailed case studies (see S1 Text). Species identification was then rechecked to obtain valid species names and remove synonyms and homonyms according to www.fishbase.org. Species presence/absence was then noted at grid cell scale using ArcGIS 10.0, and each cell covers a size of 0.5°×0.5° arc-degree. Finally 115 cells were used for further analysis, and ‘1’ or ‘0’ was scored for the occurrence or absence of a species in a grid, respectively.

#### Geology

Parameters as distance to estuary, river length, altitude (maximum, minimum and mean values), slope (maximum, minimum, mean and range values) of each cell were summarized, according to a 1:1000000 drainage map. The altitude, slope and water system data were derived originally from Shuttle Radar Topography Mission database with resolution at 3 arc-second grid and a size of 0.5°×0.5° (http://datamirror.csdb.cn). The distance of each fish point to the estuary was calculated through the Network Analyst of ArcGIS 10.0, and the mean value of distances for all fish points in a grid was calculated as the distance to estuary. The river length of a grid was the total value of all the water systems in each grid. The dataset was provided by International Scientific & Technical Data Mirror Site, Computer Network Information Center, Chinese Academy of Sciences (http://datamirror.csdb.cn).

#### Climate

Primary climate data including net primary production (NPP), air temperature (maximum, minimum and mean values) and precipitation (maximum, minimum and mean values) were gathered through the Chinese National Meteorological Information Center of China (http://cdc.cma.gov.cn/), including daily temperature and daily precipitation of 207 monitoring stations from the 1961–2000. The maximum, minimum and mean values of each parameter were calculated by comparing and averaging values of each grid within the unit through Spatial Analyst of ArcGIS 10.0. The NPP value was estimated using a climate-productivity relationship model of natural vegetation, which has been widely applied to estimate the potential productivity of zonal landscapes in China [20].

#### Palaeolake

According to published reports, originally 27 separated palaeolakes were selected and mapped on the current Yellow basin. After excluding fishes out of palaeolakes, totally 49 grid cells with fishes were left and assigned to different palaeolakes. A new matrix data containing fish presence/absence distribution in palaeolakes at grid scale was complied.

#### Glaciation

In the Quaternary China experienced the latest glacier movement, in which fish was driven southwards and then swam back after glaciation. We supposed in present time fishes in both glacier and non-glacier area could reflect the process of fish distribution, and species distributed more in glacier area could be possibly characterized by more ancestral traits. The convex hull volume, a multivariate measure derived from computational geometry, is defined as the smallest convex set enclosing the points [21, 22]. We geolocated species into the Quaternary glacial distribution map of China ([23] S1 Fig) based on their distributional information. The total of past geographical range of each species was calculated to quantify how much of each species range had been under glaciated the glacial ice-sheet (i.e. glaciated distributional area) and free of ice (i.e. non-glaciated distributional area).

### Data analysis

#### Determine the main biogeographic regions

A revised species presence-absence similarity matrix was constructed using the Bray-Curtis index, and then analyzed through group-average cluster method to illustrate the similarity of communities among sites [24]. Analyses were conducted in the statistical package Primer 5 (Primer-E, Plymouth, U.K.). Taxonomic diversity, calculated as a measure of the average taxonomic ‘distance’ between two organisms, is a modification of the Simpson index of diversity incorporating information on taxonomic relationships within a sample. Measures of taxonomic diversity were largely independent of sampling effort and thus proving increasingly invaluable in environmental assessment and conservation [25, 26]. After determining the distributional pattern, fish species presence/absence data were recompiled at divisional scale. The data matrix was then imported into Primer 5 software to measure total taxonomic diversity (TTD).

#### Partition of palaeolake, geology and climate

Considering the effects of multicolinearity among all the environmental variables, we did autocorrelation analysis to get rid of overlapping effects of all the parameters in advance. Parameters with significant statistic correlation (>0.80) were removed but retaining a main one. Finally 4 parameters as distance to estuary, all river length, mean altitude, mean slope of geology group, 3 parameters as mean NPP, mean air temperature and mean precipitation of climate group and palaeolake group were kept for further analysis, performing var-part-3groups-single-effects-FS of Variation Partitioning Analysis (VPA) under Canoco. Considering the character of presence/absence data, the raw variation and unimode method CCA were recommended by the software. Importantly, we use alphabet to distinguish the palaeolakes to avoid the effects of numerical value. We filtered the members of each group by stepwise selection to testing the simple effects. Variation partitioning was proposed a few years ago for multivariate ecological data showing spatial variation [27], to differentiate the effects of different factors in partitioning the variation in species composition. The variation of species assemblages can be decomposed into fraction Rp, the effect of palaeolake; fraction Rg, the effects of geology; fraction Rc, the effects of climate; and unexplained components (including the interactions between Rp and Rg (Rp-g), Rp and Rc (Rp-c), Rg and Rc (Rg-c), and among Rp, Rg and Rc (Rp-g-c)). Briefly, the procedure was as follows: 1), the community similarity matrix was regressed against the total set of palaeolake, geology and climate matrices to obtain the variance explained by all = Ra. 2), the community similarity matrix was regressed against the palaeolake and geology matrices to obtain the total of R(p+g) including Rp, Rg and Rp-g; the community similarity matrix was regressed against the palaeolake and climate matrices to obtain the total of R(p+c) including Rp, Rc and Rp-c; the community similarity matrix was regressed against the geology and climate matrices to obtain the total of R(g+c) including Rg, Rc and Rg-c. 3), the community similarity matrix was regressed against palaeolake, geology or climate matrix respectively to obtain independent Rp, Rg and Rc. 4), calculated the interaction fraction Rp-g = R(p+g)−Rp−Rg, Rp-c = R(p+c)−Rp−Rc, Rg-c = R(g+c)−Rg−Rc, and the Rp-g-c = Ra−Rp−Rg−Rc−Rp-g−Rp-c−Rg-c, and the unexplained variation Run = 1−Ra.

## Results

### Biogeographical pattern

**Fig 2:**
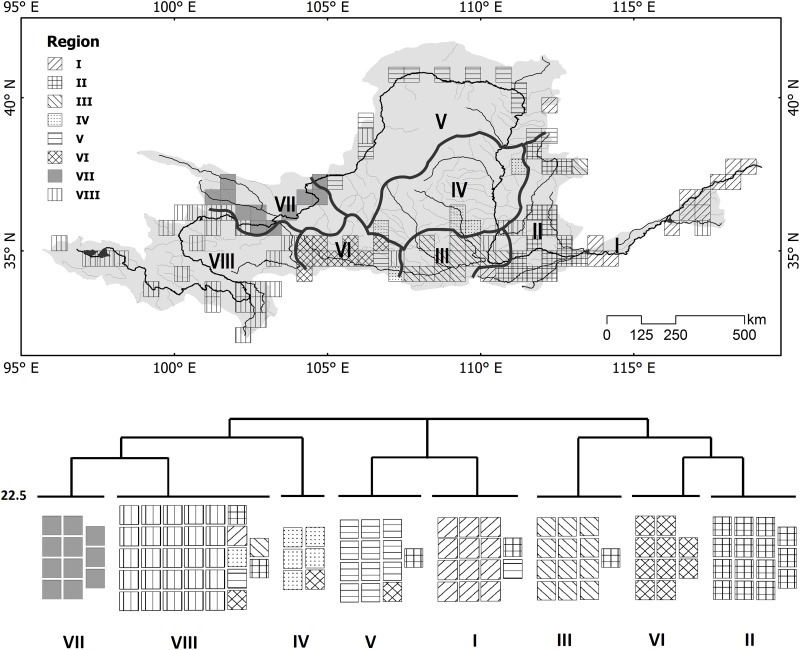
Map of the freshwater fish biogeography in the Yellow River, as well as the clustering tree. The entire basin was grouped into 8 divisions, including Region I, Estuary. The fish here are riverine species. Region II, Lower Yellow. Species from *Gobio*, Danioninae and Leuciscinae are the main fauna here. Region III, Lower Wei. The dominant species are from Danioninae, Leuciscinae, and *Triplophysa*. Region IV, Middle Yellow. Few species live in this arid area, and nearly all are from Danioninae. Region V, Upper Yellow. Fish from Cultrinae, Rhinogobio, Cobitinae and Siluridae are the dominant fauna here. Region VI, Upper Wei. Fish fauna here are dominated by *Platypharodon* and *Triplophysa*. Region VII, Huangshui. The fish here are almost entirely Schizothoracinae, *Triplophysa*, and Leuciscinae. Region VIII, Source. The fish here are all from Schizothoracinae and *Triplophysa*.

Totally 138 fish species were included in this analysis, and 111 species contributing to 80.4% of the total fishes were from the order Cypriniformes, followed by Siluriformes, 8.7% with 12 species. According to the species distribution data, the geographic patterns of the freshwater fishes in the Yellow River were divided into eight regions, corresponding to the distributions of palaeolakes at different geologic age (Fig 2), each containing special or endemic fishes (S1 Table). Regions I and II were divided by Taohuayu Gorge, a valley as a natural barrier. Though it was not deep enough to absolutely cut the migratory route between two regions, it definitely separated most species at a regional scale. The similar blocks were discovered by a series of gorges in the mainstreams and divisions of affiliated tributaries, as follows: the Jinshan Gorge shaped the eastern boundary of Region IV, and the closed flow area shaped its northern line; the Baoji Gorge obviously cut the Wei River into Region III and VI; the Wei division drew the border between Regions VI and IV, and Regions V and VII; the Qingtong Gorge divided Regions V and VII; and the Longyang and Jishi Gorges divided Regions VII and VIII.

**Fig 3:**
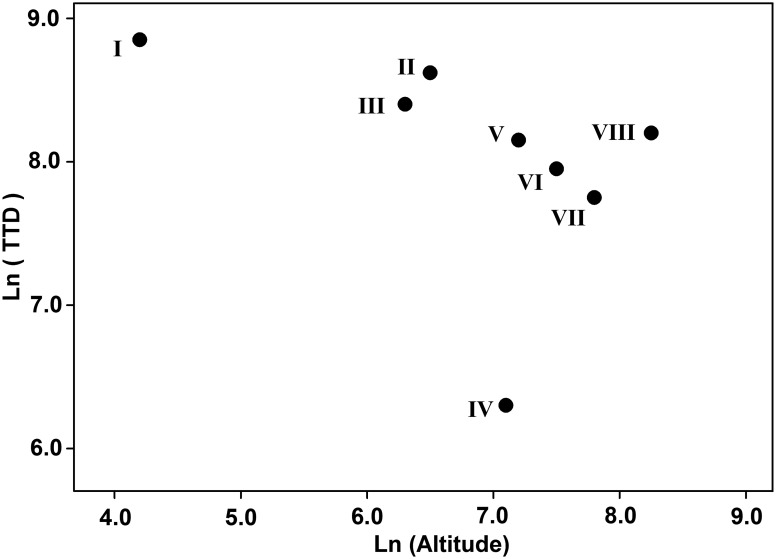
Relationship between latitude and Total Taxonomic Diversity (TTD) at logarithmic scale.

In the Yellow river, fish community of each region presented a special character of taxonomic composition (S2 Fig). Region I held the highest TTD as 7,148, significantly higher than others (p < 0.01). TTD showed a decreasing tendency from estuary to headwater except the lowest value in Region IV and a rebound in Region VIII. After excluding the outlier of Region IV, TTD showed significantly negatively linear relationship with altitude as Ln(TTD) = −0.246×Ln(Altitude)+9.951 (R^2^ = 0.760) (Fig 3).

### Partition the effects in shaping species distribution

Under VPA analysis, three groups contributed 67.3% (including 10.5% interaction) of the current fish distribution. Palaeolake explained the largest part 43.6%, significantly higher than geology group 7% and climate group 6.1% (Table 1). Interestingly, the pairwise interactions between each two of the three groups were weak (less than 2%), while the interaction among all the three groups was 8.3%. Among the other parameters, altitude and temperature respectively explained 3.9 and 3.45% of current fish biogeography.

**Table 1:** Partitioning values of explanation on fish biogeography at group and parameter scale. Rp, Rg and Rc mean the role of palaeolake, geology and climate respectively. Rp-g, interaction between palaeolake and geology; Rg-c, interaction between geology and climate; Rp-c, interaction between palaeolake and climate; Rp-g-c, interaction among palaeolake, geology and climate; ‘% of all’ = ‘% of the explained’ × (1−Run%).

Fraction	Variation	% of the Explained	% of All	DF	Mean Square	Parameter	% of Explained	% of All
PPalaeolake group (Rp)	3.683	64.7	43.6	21	0.175			
Geology group (Rg)	0.594	10.4	7.0	4	0.148	Mean Altitude	56.2	3.934
						Distance to Estuary	16.6	1.162
						Latitude	15.6	1.092
						Mean Slope	11.6	0.812
Climate group (Rc)	0.516	9.1	6.1	3	0.172	Mean Temperature	56.8	3.465
						Mean Precipitation	25.8	1.574
						Mean NPP	17.4	1.061
Rp-g	0.131	2.3	1.5					
Rg-c	-0.023	-0.4	-0.3					
Rp-c	0.087	1.5	1.0					
Rp-g-c	0.702	12.3	8.3					
Run		0	32.7					

### Fishes in both glaciated and non-glaciated areas

**Fig 4:**
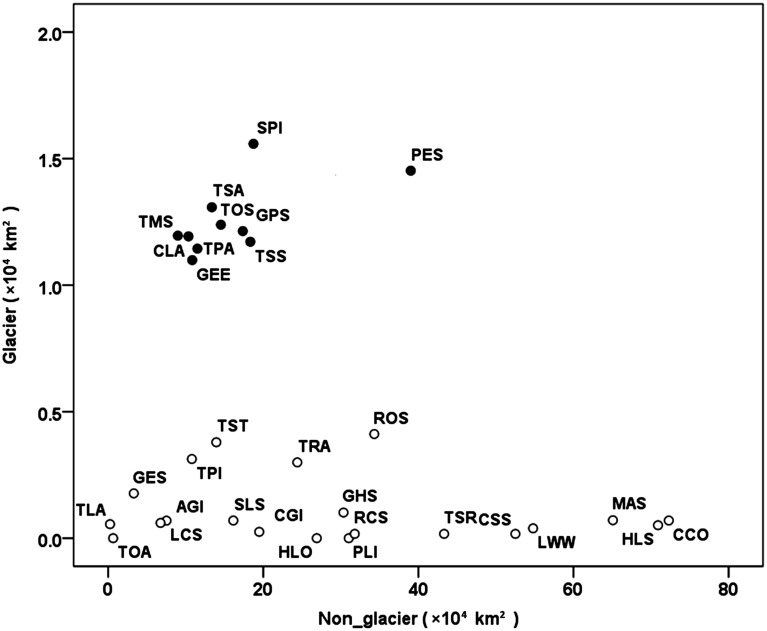
Distribution of fish in the glaciated and non-glaciated areas. The black dots are highly specialised plateau species that have a higher proportion of distributional area in glaciated areas, and the white circles are species with more ancestral characters, which are mostly found in unglaciated areas. PLI, *Phoxinus lagowskii*; LCS, *Leuciscus chuanchicus*; LWW, *Leuciscus waleckii waleckii*; HLS, *Hemiculter leucisculus*; AGI, *Acanthogobio guentheri*; HLO, *Hemibarbus labeo*; GHS, *Gobio huanghensis*; RCS, *Rhinogobio cylindricus*; CSS, *Coreius septentrionalis*; ROS, *Rhodeus ocellatus*; GPS, *Gymnodiptychus pachycheilus*; GEE, *Gymnocypris eckloni eckloni*; GES, *Gymnocypris eckloni scolistomus*; SPI, *Schizopygopsis pylzovi*; CLA, *Chuanchia labiosa*; PES, *Platypharodon extremus*; CCO, *Cyprinus carpio*; TOA, *Triplophysa obscura*; TOS, *Triplophysa orientalis*; TPI, *Triplophysa pappenheimi*; TPA, *Triplophysa pseudoscleroptera*; TRA, *Triplophysa robusta*; TSA, *Triplophysa scleroptera*; TSR, *Triplophysa sellaefer*; TSS, *Triplophysa siluroides*; TLA, *Triplophysa leptosoma*; TMS, *Triplophysa microps*; TST, *Triplophysa stenura*; CGI, *Cobitis granoei*; MAS, *Misgurnus anguillicaudatus*; SLS, *Silurus lanzhouensis*.

There were 31 species recorded in both glacier and non-glacier areas. Thirty species were from Cypriniformes, including 11 species from Genus *Triplophysa*. Another species *Silurus lanzhouensis* was from Siluridae, Siluriformes. Ten species respectively contained a range over 10,000 km^2^ glacier, showing a higher specialization to plateau habitats. For example, the species *Schizopygopsis pylzovi* and *Platypharodon extremus* respectively covered 7.7% (15,585 km^2^) and 3.6% (14,524 km^2^), of their total distributional area. The rest 21 species respectively occupied a small glacial range less than 5,000 km^2^, among which three occasional species as *Phoxinus lagowskii*, *Hemibarbus labeo* and *Triplophysa obscura* even shrank their distributions into an area of 1 km^2^ (Fig 4).

## Discussion

### Taxonomic diversity

It has been suggested that the taxonomic range of an assemblage may be important in maintaining ecosystem stability during natural or anthropogenic perturbations [28]. Taxonomic diversity is becoming more and more acceptable to explain the fish fauna for its advantage of showing characters of not only the number of species but also the taxonomy and evolution [26]. Along the Yellow river, fish composition transited from plateau species adapting to cold and rigid environment to riverine species enjoying slow current and rich nutrients. When a genus contained a higher number of species, indicating a higher species but lower genus diversion [25], e.g., in communities from upstream sites with high species richness, the fauna comprised of closely related species belonging to the genus *Triplophysa* of family Balitoridae and Schizothoracinae of family Cyprinidae, under the help of possible variety of habitats and survival requirements of species, resulted in a decreasing TTD. The similar situation also appeared in Region II. In contrast, downstream communities were typically composed of more distantly related taxon beyond species for a higher TTD, e.g., in Regions VI and VII, characterized by complicated taxonomic composition in Cyprinidae with abundant subfamilies, species differentiated into different taxonomic scale and contributed to a higher TD. Though each fish species was in different genus in Region IV, the severely scarce of fishes resulted in the lowest TTD value.

### Role of palaeolake vs. geology vs. climate in shaping fish pattern

In both temperate and tropical regions, riverine fish assemblages were found to be determined by a series of factors [29]. Many researches put attentions on the contemporary effects on fish distribution, especially climate and habitat [11, 30], while others reported that past events could draw a perceptible imprint on present spatial patterns of fish distribution and diversity [13, 31]. The Yellow River showed a unique process from scattered palaeolakes, which deemed it an interesting and distinctive process to fish biogeography.

The VPA analysis showed palaeolake was an independent factor that primarily determined the distribution of fishes in the Yellow River. Contrary to other vertebrates, fishes have higher restriction to dispersal because riverine habitat are naturally fragmented by geologic barriers (e.g., waterfalls, dry land, and watershed divides) [32, 33]. Fishes in palaeolakes showed no desire to run away except facing heavy habitat degradation, which meant the pattern of fishes was basically shaped at the beginning of palaeolake formation. Furthermore, there was a phenomenon that a certain species contains different populations in different areas [34], which would weaken the effects of palaeolake isolation when doing analysis. Besides above, mean altitude and temperature also contributed a part of the total explanation. Altitude is among the most powerful natural factors to test ecology through having a complicated affecting mechanism linking to physically atmosphere pressure, temperature, and unusual specific factors as moisture, sunshine hours, wind, season length, geology and even land use [35, 36, 37]. Water temperature acts on fish metabolism, breeding, development and growth, and behavior [38, 39]. The Yellow River winds from west to east through three terraces as Tibetan Plateau, Loess Plateau, Ningxia Plain, and North China Plain, covering a wide range of altitude and temperature. Besides the single effect by each parameter, the interactions here contributed a certain part. For example, altitude and temperature though belongs to different group, there is certainly an intrinsic relationship between them as temperature always decreases corresponding to increasing altitude.

There still were 32.7% unexplained variance in our study, which could be attributed to 1) the effects of other unscanned variables, including fish migratory trait, habitat substrate, and human activities; 2) insufficiently large spatial scales sampling; 3) the lack of abundant data; and 4) possible deficiency of identification of primary (freshwater) and endemic species.

### Process of fish biogeography under evolution of palaeolake

The family Cyprinidae firstly appeared in China in Oligocene [40, 41], and then quickly spread all over the Asian continent. The primary barbel and loaches were widely distributed in western China since Late Cretaceous. The fossil Schizothoracins appeared in Late Miocene, and in Pliocene most of them came extinct under the geomorphic uplift and was substituted by primary Schizothoracins and *Cobitis* [18, 42]. In northern China, the primary fishes were Leuciscinae and Danioninae [43, 44].

Originally, the Yellow basin was dominated by a lake-river water system developed from Tethys Sea [45], including a serial of ancient lakes and streams flowing into these lakes. During the Pleistocene epoch, a large portion of the Northern Hemisphere experienced glaciation [46, 47]. During this period, advancing and retreating glaciers moved tremendous amounts of geological surface materials, thus altering surface topography. The outcome was a distinct contrast between the homogeneous glaciated regions and the heterogeneous unglaciated landscapes. The latter provided a refugia for species recolonization following glacial retreat, exposing taxa to newly formed habitats and allowing the subsequent building of new assemblages [32]. Our analyses indicated that taxonomic fish assemblages differ among regions that have different timings of palaeolake distribution (S3 Fig).

During 1.6–1.2 Ma, the water system experienced a remarkable reorganization, portraying the basic landscape of modern Yellow basin [48], which caused the geographic differentiation among fishes. The Huangshui River separated from ancient Qinghai Lake and turned into the Datong River and then the Yellow river, forming Region VII. Fishes here contained two dominant parts, lacustrine cyprinoid and plateau species including Schizothoracinae and *Triplophysa*. The ancient Wei River further developed and flowed into ancient Tianshui Lake. Later it flowed through Baoji Gorge, and accepted the Luo River and the Jing River, forming the modern Wei River in the end [49]. Meanwhile ancient Sanmen Lake gradually shrank and disappeared [50]. Region VI (the Upper Wei) was characterized by the plateau species, while Region III (the Lower Wei) was dominated by developed Gobioninae, Cultrinae and secondary (freshwater/marine) species in Perciformes from estuary. Though the two parts contained so distinguished fauna under different environments, species *Brachymystax lenok*, which could be found in both upstream and downstream, proved the connection of the two parts, and the difference of environment also landlocked subspecies *Brachymystax lenok tsinlingensis* enjoying cold water only in Region VI. Additionally, the strong tectonic uplift caused western Sichuan Plateau tilted southeastwards, probably breaking the obstacle that limited outward diffusion of plateau fishes [18]. Under this scenario, plateau species expanded their distributional area to the southeast, and fishes enjoying the torrent in downstream also migrated upstream along with the river back to the edge of the plateau. In 0.15 Ma, the Gong River Movement entirely disconnected ancient Qinghai Lake from Yellow River [51]. The headwater further uplift and Region VIII took shape with dominance of highly specialized Schizothoracinae and *Triplophysa* [42, 52]. In spite of this, species intelligently utilized respective spaces and food, and evolved different reproductive strategies. For example, species *Chuanchia labiosa* inhabits in the upper water layer, feeding on terrestrial insects and invertebrates, and spawning in May; *Gymnocypris eckloni* in the lower layer, omnivorous and spawning in May and June; *Gymnodiptychus pachycheilus*, staying in bottom and insectivorous; *Triplophysa pappenheimi* and *Triplophysa stenura* living in rocky and current, primarily feeding on amphipoda and diatom, spawning in August and April-October, respectively [53, 54]. The movement also forced the formation of Gong River Basin by cutting through Longyang Gorge, and the upper Fen separated from Hutuo River and then connected the current lower Fen [55]. Fishes in Region II were mostly lacustrine species as Gobioninae, Acheilognathinae and Cultrinae. In 0.03–0.01 Ma, the upper and middle Yellow River further eroded headward till modern landscape developed [56, 57]. The Tibetan plateau uplift unceasingly accelerated erosion [56], and drainage density and evaporation increased [48, 58]. Region V dried out, especially after late Pleistocene 0.13 Ma. This area is a transition region containing a higher taxonomic diversity, including plateau, lacustrine and riverine fish species.

The Quaternary Ice Age caused not only a disaster to extant species but also a now-or-never chance for species evolution. We supposed that most primary species enjoying in warm water were drove to southwards, and those stayed in the cold area gradually evolved to highly specialized species, differentiated to different species with organs at different levels of evolution, such as appearance, number or shape of barbels, scales, tooth and so on, at different altitude corresponding to different stages of Tibetan Plateau uplift. Species with strong adaptability widely distributed in both glacier and non-glacier areas proved the history of fishes moving southeastward and retreating back to plateau, e.g. Cyprininae. We hypothesis the proportion of plateau species as Schizothoracinae and *Triplophysa* in glacier area to non-glacier area would probably explain the phylogenetic process, e.g., species with distributional range confined into glacier, part and non-glacier area in sequence showed a gradual specialization of ancestral characters.

In 0.01–0.003 Ma, the lower Yellow river suffered multiple river captures and diversions. For example, it flowed northwards into the Hai River then the Bohai Sea, and southwards into the Huai River then the Yellow Sea. At the end a large delta formed, supporting multiple kinds of species and now is regarded as a national wetland reserve [59]. Fishes in Region I were mostly secondary riverine species with abundant fishery resources. Species organically aggregate, respectively occupying spaces including upper, middle-lower, lower, bottom, shoal and still water, and feeding habits as omnivorous, insectivorous, detritivorous, benthic invertebrate, piscivorous and carnivorous, and spawn at different time from March to August, thus to make best use of resources and mitigate competition [60, 61, 62]. Meanwhile, after glaciations since Holocene, the geographical boundaries appeared again under climate warming; plateau fishes diffused in the Qinghai-Tibet Plateau peripheral gradually shrank back to high altitude water systems. In the 2,540 years prior to 1946 AD, the Yellow River had experienced 1,593 times flood, shifting its course 26 times noticeably and 9 times severely [63]. The serials of events left chances for contemporary climate to further consolidate the fish spatial patterns nowadays.

The Yellow River is often described as the cradle of Chinese civilization. As well as providing water for more than 155 million people and 15% of China's farmland, the river has been heavily blamed for unplanned intensive water utilization in recent years, including hydroelectric power, agriculture, industry, consequent pollution and invasion of alien species [64]. Since 1972, it has often run dry before reaching into the sea because of the decrease in rainfall and the corresponding increased use of the water resource [65]. The Yellow River Conservancy Commission had surveyed more than 13,493 km of the river in 2007 and stated 33.8% of the river system registered worse than ‘level five’ (unfit for drinking, aquaculture, industrial use, or even agriculture) according to water quality assessment standard by the UN Environmental Program [66]. Compared to the most abundant species records historically, around 30% of fish species in the river are believed to have become extinct [67, 68]. Moreover, the biogeographical pattern would definitely come to a new era in the near future, under the role of more and more intensive human activities.

## Supporting information

S1 Text References on Yellow River fishes (A) and palaeolake development (B). (DOCX)

S1 Table Endemic fish species in each biogeographic region in the Yellow River. (DOCX)

S1 Fig The distribution of glacier in the Yellow basin in the Quaternary and nowadays. (TIF)

S2 Fig Taxonomic diversity of each biogeographic division of freshwater fishes in the Yellow River. (TIF)

S3 Fig Sketch maps explaining the historical geologic events in the Yellow River basin. Palaeolakes of 3.7–2.4 Ma: 1, Mayong; 2, Ruoergai; 3, Gonghe; 4, Tongde; 5, Guide; 6, Hualong; 7, Linxia-Dongshan; 8, Qinghai; 9, Lanzhou-Minhe; 10, Lanzhou-Jingtai; 11, Tianshui; 12,Baoji; 13, Zhenyuan; 14, Huanxian; 15, Yinchuan; 16, Jilantai; 17, Linhe; 18, Hetao; 19, Baode; 20, Yangqu; 21, Taiyuan; 22, Linfen; 23, Yuncheng; 24, Samenxia; 25, Weihe; 26, Jizhong; 27, Datong. In this period there were separated lakes, and the middle reaches of Yellow River began to take shape in this period. Palaeolakes of 1.15Ma: Mayong, Ruoergai, Gonghe, Qinghai, Yinchuan, Jilantai-Hetao, Taiyuan, Fenwei, Luoyang, Jizhong, Datong. Water systems in this period: the middle and upper reaches of Yellow River, Huangshui, Datonghe, Weihe. In this period Huangshui and Datonghe changed their flow direction, forming the source of the Yellow River; the upper reaches of Weihe also changed flow direction into the Fenwei Paleolake. Palaeolakes of 0.03Ma: Mayong, Ruoergai, Qinghai, Jilantai-Hetao, Huhe. Water systems in this period: Fenhe, Hutuohe, Jinghe, Luohe. In this time most of the fossil lakes shrank and disappeared. Fenhe changed the flow direction into Yellow River; Hutuohe separated from Paleo Yellow River; Weihe, Jinghe and Luohe flowed into the Yellow River when the Fenwei Paleolake disappeared. Palaeolakes of 0.003 Ma: Zaling, Eling, Qinhai. Water systems in this period: Source of Yellow river, Paleo Yellow River delta. In this time the head erosion of Yellow River extended to the Zaling and Eling Paleolake. The middle and upper reaches of Yellow River continued to develop. The river has changed its routine many times and formed ancient Yellow River Delta; the northern part took the way of Haihe entering into the Bohai Sea and the southern part took the way of Huaihe entering into the Huanghai Sea.
(TIF)
