# Supplementary material for: Palaeolake isolation and biogeographical process of freshwater fishes in the Yellow River
Source: PLoS One. 2017 Apr 13;12(4):e0175665. doi: 10.1371/journal.pone.0175665 (PMC5391090; doi:10.1371/journal.pone.0175665)
Supplement: S1 Text — (DOCX) [file pone.0175665.s001.docx]

A:

Cai, W.X. 2013. Fishes of the Yellow River Valley. Northwest A&F University Press, Yanglin.

Chen, Y.Y., Chu, X.L., Luo, Y.L., Chen, Y.R., Liu, H.Z., He, M.J., Chen, W., Yue, P.Q., He, S.P., Lin, R.D., Cai, M.J. and Wu, B.R. 1998. Fauna Sinica (Osteichthyes): Cypriniformes II. Science Press, Beijing.

Chu, X.L., Zheng, B.S., Dai, D.Y., Huang, S.Y., Chen, Y.R., Mo, T.P., Yue, Z.H., He, M.J., Zhang, Y.F., Ma, S.M., Peng, Z.X., Wu, B.R., Ren, Z.N. and Du, J.W. 1999. Fauna Sinica (Osteichthyes): Siluriformes. Science Press, Beijing.

CTSFRYRS (Cooperation Team of Survey on Fishery Resources of the Yellow River System). 1986. Fishery Resources in Yellow River System. Liaoning Science and Technology Publishing House, Shenyang.

Wu, X.W. 1964. The cyprinid fishes of China. Vol. I. Science Press, ShanghaiScience and Technology Press, Shanghai.

Wu, X.W. 1977. The cyprinid fishes of China. Vol. II. Shanghai People’s Press, Shanghai.

Wu, Y.F. and Chen, Y. 1979. Notes on fishes from Golog and Yushu region of Qinghai Province, China. Acta Zootaxonomica Sinica 4: 287-296.

Wu, Y.F. and Wu, C.Z. 1988. Notes on fishes from the sources of the Huanghe River and Cocha Lakes, China. Acta Zootaxonomica Sinica 13: 195-200.

Wu, Y.F. and Wu, C.Z. 1992. The Fishes of the Qinghai-Xizang Plateau. Sichuan Publishing House of Science & Technology, Chengdu.

Yue, P.Q., Shan, X.H., Lin, R.D., Chu, X.L., Zhang, E., Chen, J.X., Chen, Y.F., Cao, W.X., Luo, Y.L., Chen, Y.Y., Tang, W.Q., Cai, M.J. and Wu, B.L. 2000. Fauna Sinica (Osteichthyes): Cypriniformes III. Science Press, Beijing.

Zhu, S.Q. 1995. Synopsis of Freshwater Fishes of China. Jiangsu Science and Technology Publishing House, Nanjing.

B:

Chen, F.H., Fan Y.X., Chun, X., Madsen, D.B., Oviatt, C.G., Zhao, H., Yang, L.P., Sun, Y. 2008. Preliminary study on the "Jilantai-Hetao" megalake in late Quaternary. Chinese Science Bulletin 53: 1207-1219.

Chen, F.H., Fan Y.X., Madsen, D.B., Chun, X., Zhao, H., Yang, L.P. 2008. Preliminary study on the formation mechanism of the "Jilantai-Hetao" megalake and the lake evolutionary history in Hetao region. Quaternary Sciences 28: 866-873.

Gao, M.X., Liu, S.F. 2013. Pleistocene paleosurface reconstruction and Holocen erosion quantity calculation for Guide-Gonghe-Tongde basin complex, Qinghai. Remote Sensing for Land & Resources, 25: 99-104.

Hu, X.M., Fu, J.L., Li, Y.L. 2002. Response of landform development to the tectonic movement and the climate changes in Fenhe Drainage Basin. Acta Geographica Sinica, 57: 317-324.

Ji, J.L., Zheng, H.B. Li, S.H., Huang X.T. 2006. The terraces of the Huanghe river in Pinglu county, Shanxi province and their relationship with the disappearance of the Sanmen palaeolake and the formation of the Huanghe river. Quaternary Sciences 26: 665-672.

Li, J.J., Fang, X.M., Ma, H.Z., Zhu, J.J., Pan, B.T. and Chen, H.L. 1996. Late Cenozoic landscape evolution of the upstream of the Yellow River and uplift of Qingzang Plateau. Science in China 26: 316–322.

Li, J.J, Fang, X.M, Pan, B.T., Zhao, Z.J., Song, Y.G. 2001. Late Cenozoic intensive uplift of Qinghai-Xizang Plateau and its impacts on environments in surrounding area. Quaternary Sciences 21: 381-391.

Liu, X.F. 2007. The genesis of the upper Weihe River terraces and geomorphology evolution. PhD thesis, Lanzhou University.

Lou, T.M., Du, R.H. 1960. Primary survey on the drainage development of Fenhe river graben, Shanxi, China. Acta Geographica Sinica 26(3): 154-164.

Ma, Z.Z., Han, J.Q., Xiao, F.Y. 1998. Research on serial platforms in east Pingyang paleolake. Journal of Shanxi Teachers University (Natural Science Edition) 12(2): 79-80.

Pan, B.T., Hu, Z.B., Hu, X.F., Zhang, C., Li F.Q. 2012. Time-slice of the fluvial evolution in the northern jinshaan gorge during late Cenozoic. Quaternary Sciences 32: 111-121.

Pan, B.T., Hu, Z.B., Wang, J.P., Vandenberghe, J., Hu, X.F. 2011. A magnetostratigraphic record of landscape development in the eastern Ordos Plateau, China: Transition from Late Miocene and Early Pliocene stacked sedimentation to Late Pliocene and Quaternary uplift and incision by the Yellow River. Geomorphology 125: 225-238.

Pan, B.T., Li, J.J., Cao, J.X., Chen, F.H. 1996. Study on the geomorphic evolution and developmentofthe Yellow river in the Hualong Basin. Mountain Research14: 153-158.

Pan, B.T., Liu, X.F., Gao, H.S., Wang, Y., Li, J.J. 2007. Paleomagnetic dating of the terraces in Longxi,the upstream of Weihe river, and its cause. Progress in Natural Science, 17: 1063-1068.

Tian, C.L., Li, D.T., Liu, T.Q. 1990. An investigation into the characteristics of the sedimentary filling In Es~(2-3), Jizhong Basin. Acta Sedimentologica Sinica 8(4): 33-40.

Wang, S.M., Wu, X.H., Zhang, Z.K., Jiang, F.C., Xue, B., Tong, G.B., Tian, G.Q. 2001. Environmental change of Sanmen paleolake sedementary records and the Yellow River connection to eastwards flowing. Science in China 31: 760-768.

Wang, Y.F., Wang, S.M., Xue, B., Ji, L., Wu, J.L., Xia, W.L., Pan, H.X., Zhang, P.Z., Chen, F.H. 1995. Sedimentology basis on the Yellow River capturing Roergai paleolake. Chinese Science Bulletin 40: 723-725.

Wu, Z.Y., Ren, D.C., Zhang, R.Y. 1998. The Yellow River Annals II: The Yellow River Basin. Zhenzhou: Henan People’s Press.

Xu, M.Q. 1988. An outline of the Quaternary environmental evolution of the Roergai Plateau in northwestern Sichuan. Journal of Southwest Teachers University 32(4): 94-100.

Yuan, B.Y., Chen, K.Z., Bowler, J.M., Ye, S.J. 1990. The formation and evolution of the qinghai lake. Quaternary Sciences 10: 233-243.

Yuan, B.Y., Wang, Z.H. 1995. Uplift of the Qinghai-Xizang Plateau and the Yellow River physiographic period. Quaternary Sciences: 353-359.

Zhang, Z.Y., Yu, Q.W., Zhang, K.X., Gu, Y.S., Xiang, S.Y. 2003. Geomorphological evolution of quaternary river from upper yellow river and geomorphological evolution investigation for 1:250,000 scale geological mapping in Qinghai-Tibet plateau. Earth Science-Journal of China University of Geosciences 28: 621-626, 633.

Zhu, Z. 1989. The formation of river terraces and evolutionof drainage system in the middle Yellow river. Acta Geographica Sinica 44: 429-440.
