# Supplementary material for: Palaeolake isolation and biogeographical process of freshwater fishes in the Yellow River
Source: PLoS One. 2017 Apr 13;12(4):e0175665. doi: 10.1371/journal.pone.0175665 (PMC5391090; doi:10.1371/journal.pone.0175665)
Supplement: S1 Table — (DOCX) [file pone.0175665.s002.docx]

| Region | Species | Family | Order |
| --- | --- | --- | --- |
| I | *Rhinogobio typus* | Cyprinidae | Cypriniformes |
|  | *Elopichthys bambusa* | Cyprinidae | Cypriniformes |
|  | *Culter dabryi* | Cyprinidae | Cypriniformes |
|  | *Toxabramis swinhonis* | Cyprinidae | Cypriniformes |
|  | *Pseudolaubuca sinensis* | Cyprinidae | Cypriniformes |
|  | *Xenocypris microlepis* | Cyprinidae | Cypriniformes |
|  | *Lefua costata* | Balitoridae | Cypriniformes |
|  | *Pseudobagrus truncates* | Bagridae | Siluriformes |
| II | *Aphyocypris chinensis* | Cyprinidae | Cypriniformes |
| III | *Distoechodon tumirostris* | Cyprinidae | Cypriniformes |
|  | *Gobio meridionalis* | Cyprinidae | Cypriniformes |
|  | *Phoxinus oxycephalus* | Cyprinidae | Cypriniformes |
|  | *Barbatula nuda* | Balitoridae | Cypriniformes |
|  | *Triplophysa bleekeri* | Balitoridae | Cypriniformes |
|  | *Triplophysa heyangensis* | Balitoridae | Cypriniformes |
| V | *Leuciscus chuanchicus* | Cyprinidae | Cypriniformes |
|  | *Platypharodon extremus* | Cyprinidae | Cypriniformes |
| VI | *Brachymystax lenok tsinlingensis* | Salmonidae | Salmoniformes |
| VIII | *Chuanchia labiosa* | Cyprinidae | Cypriniformes |
|  | *Gymnocypris eckloni scolistomus* | Cyprinidae | Cypriniformes |
|  | *Triplophysa alticeps* | Balitoridae | Cypriniformes |
|  | *Triplophysa obscura* | Balitoridae | Cypriniformes |
|  | *Triplophysa brevicauda* | Balitoridae | Cypriniformes |
|  | *Triplophysa leptosome* | Balitoridae | Cypriniformes |
|  | *Triplophysa longianguis* | Balitoridae | Cypriniformes |
