# Supplementary figures and images for: Palaeolake isolation and biogeographical process of freshwater fishes in the Yellow River
Source: PLoS One. 2017 Apr 13;12(4):e0175665. doi: 10.1371/journal.pone.0175665 (PMC5391090; doi:10.1371/journal.pone.0175665)

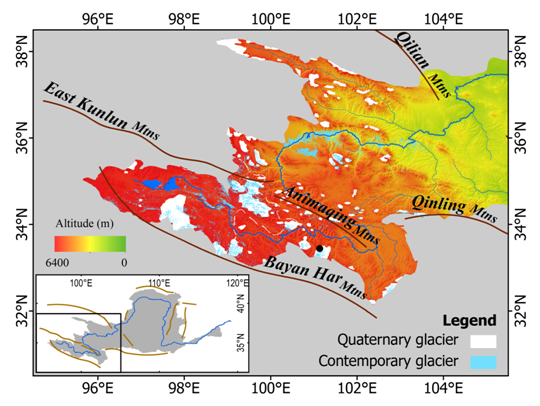

Supplement: S1 Fig — (TIF) [file pone.0175665.s003.tif]

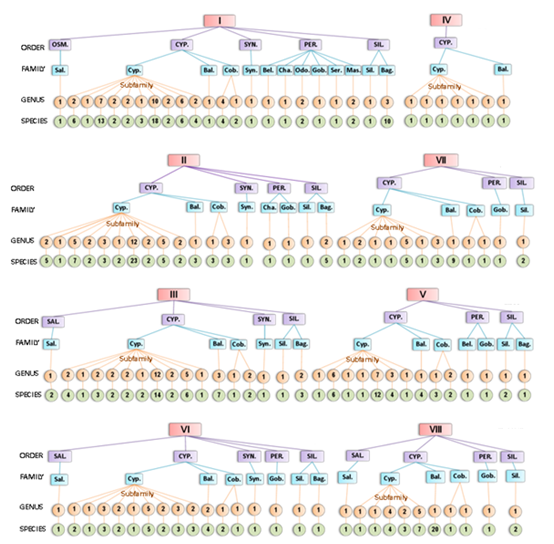

Supplement: S2 Fig — (TIF) [file pone.0175665.s004.tif]

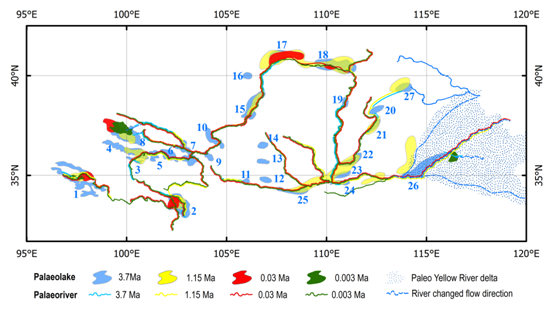

Supplement: S3 Fig — Palaeolakes of 3.7–2.4 Ma: 1, Mayong; 2, Ruoergai; 3, Gonghe; 4, Tongde; 5, Guide; 6, Hualong; 7, Linxia-Dongshan; 8, Qinghai; 9, Lanzhou-Minhe; 10, Lanzhou-Jingtai; 11, Tianshui; 12,Baoji; 13, Zhenyuan; 14, Huanxian; 15, Yinchuan; 16, Jilantai; 17, Linhe; 18, Hetao; 19, Baode; 20, Yangqu; 21, Taiyuan; 22, Linfen; 23, Yuncheng; 24, Samenxia; 25, Weihe; 26, Jizhong; 27, Datong. In this period there were separated lakes, and the middle reaches of Yellow River began to take shape in this period. Palaeolakes of 1.15Ma: Mayong, Ruoergai, Gonghe, Qinghai, Yinchuan, Jilantai-Hetao, Taiyuan, Fenwei, Luoyang, Jizhong, Datong. Water systems in this period: the middle and upper reaches of Yellow River, Huangshui, Datonghe, Weihe. In this period Huangshui and Datonghe changed their flow direction, forming the source of the Yellow River; the upper reaches of Weihe also changed flow direction into the Fenwei Paleolake. Palaeolakes of 0.03Ma: Mayong, Ruoergai, Qinghai, Jilantai-Hetao, Huhe. Water systems in this period: Fenhe, Hutuohe, Jinghe, Luohe. In this time most of the fossil lakes shrank and disappeared. Fenhe changed the flow direction into Yellow River; Hutuohe separated from Paleo Yellow River; Weihe, Jinghe and Luohe flowed into the Yellow River when the Fenwei Paleolake disappeared. Palaeolakes of 0.003 Ma: Zaling, Eling, Qinhai. Water systems in this period: Source of Yellow river, Paleo Yellow River delta. In this time the head erosion of Yellow River extended to the Zaling and Eling Paleolake. The middle and upper reaches of Yellow River continued to develop. The river has changed its routine many times and formed ancient Yellow River Delta; the northern part took the way of Haihe entering into the Bohai Sea and the southern part took the way of Huaihe entering into the Huanghai Sea. (TIF) [file pone.0175665.s005.tif]
